# Case report: Difficult diagnosis of *Mycobacterium tuberculosis* infection in patients after allogeneic hematopoietic stem cell transplantation: two case reports and a literature review

**DOI:** 10.3389/fonc.2024.1283320

**Published:** 2024-05-28

**Authors:** Zhenghua Liu, Dali Cai, Nan Su

**Affiliations:** Department of Hematology, The First Hospital of China Medical University, Shenyang, Liaoning, China

**Keywords:** MTB (*Mycobacterium tuberculosis*), mNGS (metagenomic next-generation sequencing), allo-HSCT, diagnosis, case report

## Abstract

**Background:**

*Mycobacterium tuberculosis* (MTB) is a relatively infrequent infection encountered during hematopoietic stem-cell transplantation (HSCT). The identification of MTB following HSCT remains a complex task, with delayed detection and misdiagnosis potentially resulting in unfavorable outcomes. Metagenomic next-generation sequencing (mNGS) represents a novel, highly sensitive, and rapid diagnostic tool in clinical settings for discerning intricate infections and detecting exceedingly rare pathogens

**Methods:**

With the aid of mNGS, we diagnosed MTB in the lymph nodes and lungs of two patients with hematological diseases following allogeneic peripheral blood hematopoietic stem cell transplantation. Both patients presented with a fever, localized symptoms, and clinical signs. Following inconclusive results from routine tests, impractical biopsy procedures, and unsuccessful responses to empirical treatments, mNGS was employed as a final recourse, revealing DNA fragments of MTB in blood samples.

**Results:**

The diagnoses were ultimately confirmed in conjunction with additional clinical evidence. The application of mNGS in MTB cases after allogeneic HSCT has rarely been reported. The mNGS technique can provide a prompt and highly sensitive indication leading to the definitive diagnosis of MTB in complex post-transplant scenarios.

## 1 Introduction

Due to transient or persistent deficiency in cellular and humoral immunity, recipients of hematopoietic stem cell transplantation (HSCT) are often vulnerable to infections caused by opportunistic and foreign microbes, especially in allogeneic HSCT. The incidence of *Mycobacterium tuberculosis* (MTB) in HSCT recipients varies, ranging from 0.4% in nonendemic areas to approximately 16% in endemic areas, which is higher than that in immunocompetent populations in the same region (1–4). Several retrospective analyses have indicated that HSCT recipients face a heightened susceptibility to MTB infection, especially in the presence of factors such as mismatched HLA transplantation, acute or chronic graft-versus-host disease (GVHD), and total body irradiation (TBI) (2, 5).

Due to atypical and nonspecific clinical presentations (2), along with the limitations of less sensitive tests and the impracticality of biopsy procedures due to associated risks such as bleeding and other complications, the diagnosis of MTB remains challenging in patients following allogeneic HSCT. A novel technique known as metagenomic next-generation sequencing (mNGS) has emerged as a sensitive and rapid method for detecting DNA and RNA fragments of various microorganisms present in clinically suspected samples without the need for *in vitro* culturing and amplification. This advanced approach has been increasingly utilized in cases of complex infections where conventional clinical microbiological assays have proven ineffective (6). In the context of two reported cases of MTB infection following allogeneic HSCT, the diagnosis heavily relied on the application of mNGS technology.

## 2 Transplantation protocol and mNGS protocol

### 2.1 Transplantation protocol

Case 1 was diagnosed with acute myeloid leukemia (AML) with myelodysplasia-related changes, while case 2 had classical Hodgkin’s lymphoma. Both cases underwent peripheral stem cell transplantation from fathers who were five of 10 human leukocyte antigen (HLA)-matched. The conditioning regimen for case 1 was busulfan, fludarabine, cytarabine (BFA), which included busulfan 0.8 mg/kg every 6 h from days −8 to −5, fludarabine 30 mg/m^2^ daily from days −8 to −4, and cytarabine 2 g/m^2^ daily from days −8 to −4. Case 2 received a conditioning regimen of busulfan, fludarabine, melphalan (BFM), which consisted of busulfan 0.8 mg/kg every 6 h from days −9 to −7, melphalan 50 mg/m^2^ daily from days −7 to −6, and fludarabine 30 mg/m^2^ daily from days −6 to −2. The protocol for GVHD prophylaxis involved four drugs following the Beijing mode for haploidentical HSCT: Cyclosporine A was administered at a dose of 3 mg/kg/day via continuous infusion over 24 h from day −1 to achieve a targeted therapeutic concentration ranging from 200 to 300 ng/mL; short-term methotrexate (MTX) was initiated at 15 mg/m^2^/day on day +1 and 10 mg/m^2^/day on days +3, +6, and +11; mycophenolate mofetil (MMF) was taken orally at 1.0 g twice daily from days +1 to +30, with a gradual tapering off until day +60 if no acute GVHD occurred, and rabbit antihuman thymocyte globulin (r-ATG, Genzyme Polyclonals S.A.S, France) was given at 2.5 mg/kg on days −5 and −2.

### 2.2 mNGS protocol

Blood samples were collected from patients following the requirements for mNGS testing and storage. Nucleic acid extraction from blood samples was fragmented to yield 150-bp to 200-bp fragments, and DNA fragments were constructed using an end-repair method. The MGISEQ-2000 platform was used for sequencing by BGI Patho-Genesis Pharmaceutical Technology Co. Ltd, China. The low-quality data and data less than 35 base pairs in length were removed to obtain high-quality sequencing results. The human nucleic acid sequence data were removed using Burrows–Wheeler Aligner (BWA) software alignment. The remaining data were compared with a dedicated microbial database downloaded from NCBI after removing low-complexity sequences. The sequenced data were then classified and arranged according to viruses, bacteria, fungi, and parasites.

## 3 Cases

### 3.1 Case 1

A 25-year-old man was diagnosed with AML with myelodysplasia-related changes. He was treated with cytarabine and daunorubicin before undergoing transplantation. Prior to transplantation, various tests, such as T-SPOT (The interferon gamma release assay), lung CT, and abdominal CT, were conducted to rule out evidence of tuberculosis infection. He received granulocyte colony-stimulating factor-mobilized peripheral blood stem cells (PBSCs) from his biological father after a myeloablative conditioning regimen, including CD34^+^ cells at 4.8 × 10^6^/kg and CD3^+^ cells at 4.7 × 10^8^/kg. The GVHD prophylaxis was administered as previously mentioned. Neutropenic fever was managed effectively with meropenem during the neutropenic period. Neutrophil and platelet engraftment were achieved on days +14 and +13, respectively. Subsequently, the patient experienced fever and abdominal pain from day +26. A lung CT showed only small nodules in both lungs and faint shadows in the lower lobe of the left lung. No typical imaging findings of tuberculosis were found. Isolated peritoneal multiple enlarged lymph nodes were identified through contrast abdominal CT scan and B-ultrasonography. The maximal size of lymph nodes was 1.57 cm × 1.08 cm, without the involvement of superficial lymph nodes. Potential causes of fever and lymphadenopathy, such as post-transplant lymphoproliferative disease, were ruled out, and routine examinations for MTB were negative. After unsuccessful empirical anti-infection therapies and the impracticality of performing a biopsy on peritoneal lymph nodes, mNGS was attempted. During episodes of fever, 5 mL of blood was collected and analyzed for the presence of MTB DNA fragments. Subsequently, experimental anti-MTB treatment with isoniazid, rifampin, and pyrazinamide was initiated, resulting in the resolution of fever and abdominal pain after 2 weeks. Shrunken lymph nodes were noticed on a CT scan during a later follow-up. A tentative diagnosis of MTB was confirmed. Cyclosporine A was gradually tapered on day +120 and discontinued by day +180 after allogeneic HSCT. The patient experienced mild and localized chronic GVHD post-HSCT without systemic administration of corticosteroids. Anti-MTB therapies were continued until 1 year after HSCT. Presently, over 3 years have passed, and the patient has resumed normal activities without any further complications or recurrence of MTB.

### 3.2 Case 2

**Figure 1:**
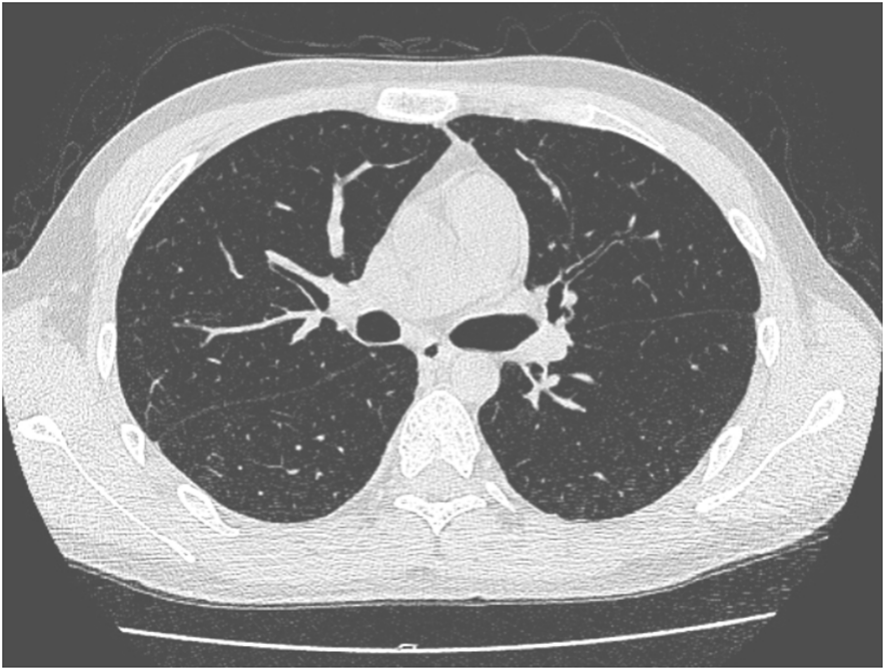
Case 2 lung CT on day −18.

**Figure 2:**
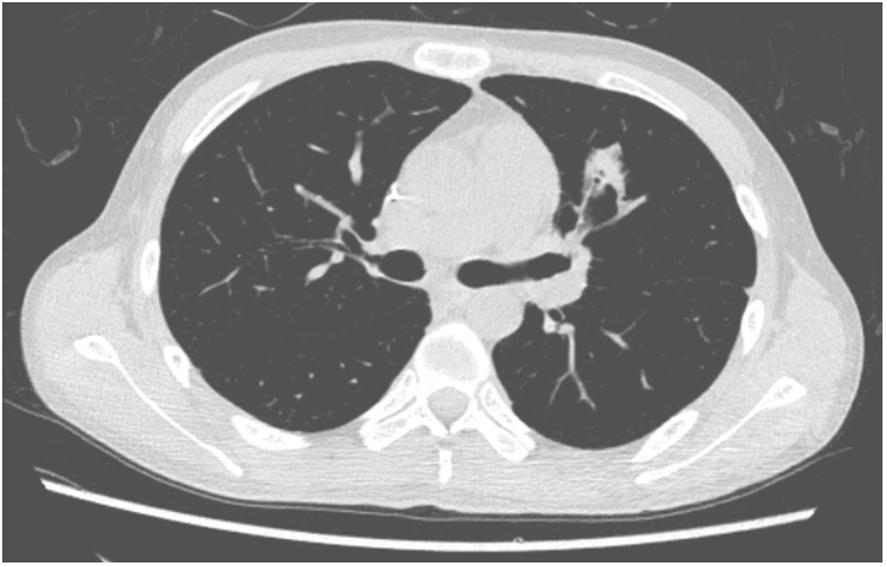
Case 2 lung CT on day +31.

**Figure 3:**
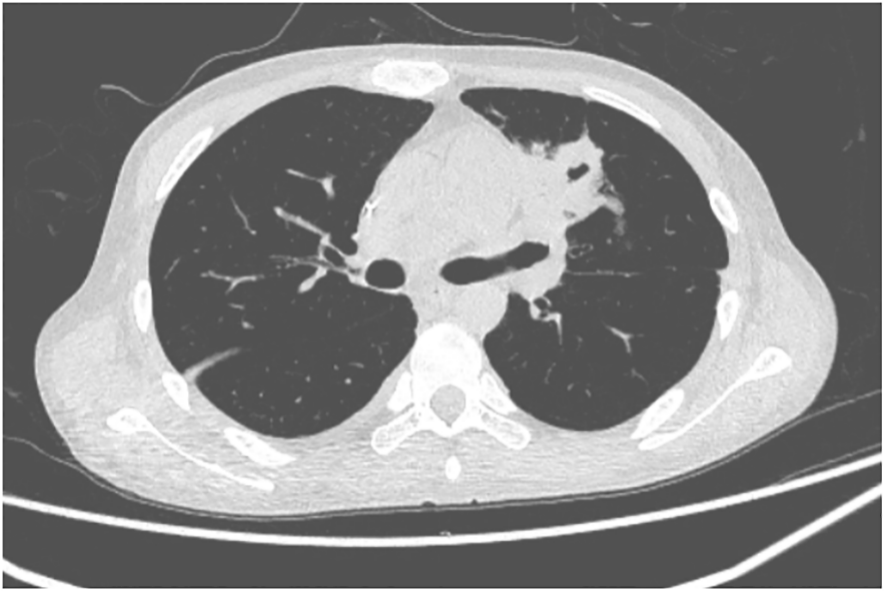
Case 2 lung CT on day +48.

A 27-year-old man has been diagnosed with classical Hodgkin’s lymphoma IIA (IPSS). He received ABVD, ICE, DECP, GDP regimens, and autologous transplantation. However, the disease recurred 5 months after autologous transplantation and recurred again after achieving remission with PD-1 inhibitor treatment. Later, he was treated with decitabine in combination with sindilizumab, a CD30 monoclonal antibody combined with bendamustine, and bendamustine as monotherapy. He had a negative T-SPOT result before transplantation. Ultimately, he underwent haploidentical HSCT following a reduced dose conditioning regimen BFM upon achieving complete remission with CD30 monoclonal antibody and bendamustine, with consolidation using the same regimen until the washout period of the PD1 inhibitor reached 90 days. The G-CSF-mobilized PBSCs from his biological father were infused with CD34^+^ cells at 3.7 × 10^6^/kg and CD3^+^ cells at 5.7 × 10^8^/kg. The GVHD prophylaxis was intensified with a reduced dose of cyclophosphamide at 14.5 mg/kg/day on days +3 and +4 post-transplant, in addition to cyclosporine A, MTX, MMF, and ATG, as reported by Wang (7). Neutrophil engraftment was achieved on day +15, but the platelet graft failed. On day +30, he developed a fever and cough. A lung CT scan revealed a local opacity in the upper lobe of the left lung, which was different from the appearance observed in the pretransplant lung CT scan (**Figures 1**–**3**). Empirical anti-infection therapies were immediately initiated, including antibiotics, while screening was done for bacteria, fungi, viruses, and parasites to identify the underlying cause. Consequently, due to the lack of positive results from the laboratory, poor response to the treatment, infeasibility of bronchoalveolar lavage (BAL) due to poor condition, and low platelet count, we conducted mNGS again to scan for pathogens in the blood samples. A DNA fragment of MTB was identified on day +45 and subsequently examined for its presence in sputum samples. Finally, MTB was successfully isolated on day +50.

Since the probable diagnosis of pulmonary tuberculosis (TB) was established, anti-MTB therapy was initiated with isoniazid, rifampin, and pyrazinamide. As a result, the body temperature dropped. Unfortunately, on day +46, the patient developed transplant-associated thrombotic microangiopathy (TA-TMA), characterized by the presence of protein in the urine, hypertension, heavy reliance on red blood cell (RBC) and platelet transfusions, elevated blood LDH levels, and the presence of schistocytes. Discontinuation of cyclosporine A, initiation of eculizumab (an antibody against complement C5), and plasma exchange therapy were all attempted, but the patient succumbed to multiorgan failure.

## 4 Discussion

Research has indicated that changes in Th1 cell response in hematological diseases, either due to the diseases themselves, anti-hematologic tumor treatments, or hematopoietic stem cell transplantation (often involving the use of high doses of corticosteroids), may result in compromised immune function. This compromised immunity is a significant factor in the progression from latent TB infection to active TB (8). After undergoing allogeneic HSCT, individuals with persistent cellular immunodeficiency are susceptible to various pathogens, such as bacteria, fungi, viruses, and parasites. In this particular case study, two instances of MTB infection were diagnosed using mNGS after conventional screenings failed to detect MTB infection, underscoring the diagnostic utility of mNGS in complex scenarios post-allogeneic HSCT. So far, no similar reports have been documented.

The identification of active MTB infection after transplantation remains a complex task (1). One primary challenge is the reduced specificity of diagnosis due to exposure to broad-spectrum antibiotics aimed at preventing or managing common bacterial infections. Additionally, complications may arise if the individual is coinfected with other microorganisms after HSCT. Another significant hurdle is the limited availability of sensitive and specific diagnostic tools for TB, which are crucial for accurate diagnosis. While TB culture is considered the gold standard, its sensitivity is relatively low, and the process is time-consuming (**Table 1**). Immunological methods are often unsuitable for HSCT patients undergoing immunosuppressive therapy. Targeted polymerase chain reaction (PCR) methods have been developed to adapt to the evolving nature of the disease, including its epidemiology and global resistance patterns (11, 16). Notably, advanced diagnostic tools such as X-pert MTB/RIF and the highly sensitive but less popular Xpert MTB/RIF Ultra, recommended by the World Health Organization (WHO), were not accessible to the patients in this study. Furthermore, the risk of bleeding or poor performance after HSCT can impede the feasibility of conducting biopsies. Consequently, delays in diagnosing clinically active TB infections are common, leading to unfavorable outcomes and increased mortality rates (20).

**Table 1:** The advantages and limitations of typical diagnostic methods for *Mycobacterium tuberculosis*.

Detections	Advantages	Limitations
Microscopy smear	Low cost	Hard to distinguish between *Mycobacterium tuberculosis* and nontuberculous mycobacteria (9)
	Rapidity (10)	Low sensitivity and poor positive rate (11)
Culturing of mycobacteria	Widely used	Time-consuming (12)
	Low cost	Low sensitivity and poor positive rate
	Gold standard	Production of harmful aerosols (13)
Xpert MTB/RIF	High cost	Analysis limited to specific mutations for few antibiotics (14, 15)
	Rapidity (10)	Lack of detection of heteroresistance (16)
	Drug resistance detection (17)	
	High sensitivity and specificity (14)	
mNGS	Detection of numerous mutations for a large panel of antibiotics	Expensive
	Detection of heteroresistance (16)	Time-consuming
	High sensitivity in pulmonary and extra-pulmonary samples, especially in MTB meningitis (18, 19)	Sequencing certain parts of genome (16)
	Identifying all pathogens	Interference of noncausative pathogens
WGS	Detection of all putative mutations for all antibiotics	Expensive
	Sequencing the entire genome	Time-consuming

Currently, there is an urgent demand for more sensitive and reliable diagnostic tools. Metagenomic next-generation sequencing is a comprehensive method that involves sequencing entire microbial nucleic acid fragments in suspected samples and analyzing and comparing them with a microbiome database to detect potential microbial species. This method offers several advantages and disadvantages (6, 21). It retrieves all DNA without bias and is a sensitive, specific, and rapid method for detecting pathogens present in clinical samples without the need for *in vitro* culture or amplification. It reveals the true status of all copathogens in suspected samples without any data loss, particularly for slowly and poorly growing pathogens *in vitro*. It can recognize both known and unknown pathogens without requiring specific conditions, like particular primers for PCR. It can detect uncommon pathogens and microbes within human cells, such as MTB, and contributes as a supplementary tool in diagnosis by providing a clue to providing a definitive diagnosis. In comparison with Xpert MTB/RIF, mNGS exhibits higher sensitivity in detecting pulmonary and extra-pulmonary samples, particularly in cases of MTB meningitis (18, 19). For complex infections caused by multiple pathogens, mNGS could identify all pathogens simultaneously, preventing the oversight of other causative microbes subsequent to the identification of MTB with Xpert MTB/RIF. However, the disadvantages of mNGS include background noise from the human DNA genome, interference from noncausative pathogens, the absence of uniform standards for the entire procedure, and the standardization of the bioinformatics analysis process. Therefore, combined with the MTB culture and Xpert MTB/RIF, it has the potential to significantly enhance diagnostic efficacy.

The concentration of MTB in blood samples is lower compared to sputum samples for pulmonary MTB. Despite this, blood samples are preferred over sputum samples for mNGS due to reduced microbial background interference and to avoid using unqualified sputum samples. Moreover, mNGS with high sensitivity could compensate for the low numbers of DNA copies in the blood samples (18). Certainly, it is imperative to meticulously analyze the positive outcomes to ascertain their relevance to the clinical context. For instance, in case 2, initial attempts to isolate MTB from sputum yielded negative results. After persistent efforts, we finally achieved the target of detecting MTB in blood using mNGS. In case 1, diagnosing isolated lymph node tuberculosis proved challenging due to the patient’s poor post-allo-HSCT condition, which did not allow for safe invasive and histological procedures. Metagenomic next-generation sequencing of blood samples finally identified MTB DNA, validated by the treatment outcome and clinical matches. Studies have shown that mNGS is more suitable when the patient presents with unexplained manifestations beyond traditional assays or atypical symptoms, such as fever, dyspnea, and elevated inflammatory markers. It is also recommended when there is a strong suspicion of a multipathogen infection, in cases where the patient is in critical condition, and when timely and comprehensive detection results are crucially needed (22).

Various innovative diagnostic techniques for tuberculosis have been introduced. For instance, researchers are exploring the potential use of tongue swabs as an alternative to collecting sputum for diagnosing TB. The authors argue that while molecular diagnostics for TB are highly sensitive, their feasibility is constrained in resource-limited settings due to expensive equipment and inadequate infrastructure. Additionally, collecting sputum for TB diagnosis presents challenges and risks associated with the production of harmful aerosols. The study provides evidence supporting the possibility of detecting TB from a tongue swab without the requirement for DNA extraction or purification processes (13). Whole-genome sequencing (WGS) is widely recognized as the global benchmark for characterizing MTB isolates (23–25). WGS demonstrates exceptional discriminatory capability and offers a comprehensive approach to anticipating antibiotic resistance based on genotype. Nevertheless, additional clinical validation is necessary for these methods.

MTB infection after allo-HSCT is relatively less common than in solid organ transplantation (1, 2). This disparity may be attributed in part to the administration of antibiotics during the neutropenic phase of transplantation, especially fluoroquinolone, which is routinely used as prophylaxis for bacterial infection and actually has potential ability against MTB (2, 26, 27). However, neither of the two cases in our report received fluoroquinolones for various reasons. The diagnosis of MTB infection in our report was established on days +30 and +50, respectively, which is shorter than the timelines reported in most reports (2, 3). The reason is probably related to the endogenous reactivation of MTB (2), and there is no infection prophylaxis with fluoroquinolone during neutropenia. The high sensitivity of mNGS may provide an early indication for the prompt confirmation of MTB diagnosis. However, this case report is still limited due to the small number of cases, and it would be preferable to have a larger sample size to validate this technique in order to demonstrate that the presence of positive DNA in the blood truly reflects active infection rather than latent infection.

## 5 Conclusions

In conclusion, the diagnosis of MTB post-HSCT is still difficult, and mNGS might be an important technique for complicated infections, supplying important clues to the final diagnosis.

## Data availability statement

The original contributions presented in the study are included in the article/supplementary material. Further inquiries can be directed to the corresponding authors.

## Ethics statement

Ethical review and approval was not required for the study on human participants in accordance with the local legislation and institutional requirements. The studies were conducted in accordance with the local legislation and institutional requirements. The participants provided their written informed consent to participate in this study. Written informed consent was obtained from the individual(s) for the publication of any potentially identifiable images or data included in this article.

## Author contributions

ZL: Writing – original draft. DC: Writing – review & editing. NS: Writing – review & editing.
